# Gut microbiota composition in preterm infants with and without necrotizing enterocolitis: a systematic review and narrative synthesis

**DOI:** 10.1099/acmi.0.001077.v3

**Published:** 2025-12-19

**Authors:** Fatemah Sadeghpour Heravi, Gerald L. Murray, Jennifer A. Danielewski, Suzanne M. Garland, Erica L. Plummer

**Affiliations:** 1 Department of Obstetrics, Gynaecology and Newborn Health, The University of Melbourne, Parkville, Australia; 2 Murdoch Children’s Research Institute, Parkville, Australia; 3 Women’s Centre for Infectious Diseases, The Royal Women’s Hospital, Parkville, Australia; 4 School of Translational Medicine, Monash University, Melbourne, Australia

**Keywords:** *Clostridium*, *Enterobacteriaceae*, gut microbiota, necrotizing enterocolitis, preterm, Proteobacteria

## Abstract

Necrotizing enterocolitis (NEC) is a devastating gastrointestinal disorder in preterm infants with a high mortality rate. The aetiology of NEC appears to be multifaceted; however, gut microbiota dysbiosis likely plays a significant role. This systematic review aimed to describe how the gut microbiota of preterm infants with NEC differs from infants without NEC (PROSPERO: CRD42022344126). Databases were searched from inception to 22 June 2022 to identify eligible studies that examined the gut microbiota composition of preterm infants with and without NEC using sequencing methods. Results were described narratively. We identified 28 eligible studies. Overall, findings were heterogeneous and no single gut microbiota signature was associated with NEC in all studies. Importantly, 3 studies reported no difference in the gut microbiota composition between NEC and healthy infants, while studies reported a difference using one or more analytical method (i.e. alpha diversity, beta diversity or differential abundance analysis). Of note, NEC (or development of NEC) was positively associated with increased detection and/or abundance of *Enterobacteriaceae* (*n*=11 studies), *Clostridium* (*n*=8) and Proteobacteria (*n*=2). The taxa most frequently associated with NEC (*Enterobacteriaceae*, *Clostridium*, and Proteobacteria) may play an important role in the pathogenesis of NEC and should be further explored.

## Data Summary

The authors confirm that all supporting data have been provided within the article or through supplementary data files.

## Introduction

Necrotizing enterocolitis (NEC) is a serious gastrointestinal disorder that affects preterm infants and is characterized by inflammation and necrosis of the intestinal tissue [1]. NEC severity is determined by the Bell staging criteria, which categorize NEC into stages, with stage III being associated with the most severe clinical signs [2,3]. The incidence of NEC varies depending on the population and the definition used. In general, the reported incidence ranges from 2 to 12% among premature infants, with higher rates reported in extremely premature infants [4].

The aetiology of NEC is not known, but it is considered to be a multifactorial and complex disease. Notably, an imbalanced gut microbiota (dysbiosis) has been hypothesized to contribute to the development of NEC among infants born prematurely [5]. It has been reported that the gut microbiota of preterm infants is distinct from that of full-term infants, characterized by an increased proportion of potentially pathogenic bacteria and decreased diversity [6]. Disrupted gut microbiota composition may lead to an increase in gut permeability, inflammation and oxidative stress, which in turn may contribute to the development of NEC [7]. Numerous studies have examined the association between NEC and gut microbiome composition. For example, some studies have demonstrated a positive correlation between NEC and high abundance of Gram-negative bacteria (such as *Enterobacteriaceae*), whereas others have associated NEC with low abundance of anaerobic bacteria such as *Bifidobacterium* and reduced microbial diversity [8,10]. In 2017, Pammi *et al.* re-analysed 16S rRNA gene sequencing data from eight culture-independent studies and reported that NEC was preceded by a microbial dysbiosis, characterized by increased abundances of Proteobacteria and decreased relative abundances of Firmicutes and Bacteroidetes [11].

Given the increasing evidence linking gut microbiota to NEC, it is critical to examine this association in a systematic manner. By incorporating the highest number of studies to date, this systematic review aimed to synthesize current published evidence and critically evaluate potential sources of bias to provide a comprehensive understanding of how the gut microbiome of preterm infants with NEC differs from that of preterm infants without NEC.

## Methods

This review was conducted and reported according to the guidelines set out by the Preferred Reporting Items for Systematic Reviews and Meta-analyses [PRISMA; (Supplementary Material 1)] [12] and the review protocol was prospectively registered with PROSPERO (CRD42022344126).

### Searching strategy

Databases [MEDLINE (PubMed) and Embase] were searched from inception to 22 June 2022 using the following keywords: ‘necrotizing enterocolitis’, ‘preterm infant’ and ‘gut microbiota’. Full search strings are provided in File S1, available in the online Supplementary Material. Reference lists of included studies and prior systematic reviews on the topic were also screened for eligibility. We did not search grey literature.

### Selection criteria and screening

Studies were uploaded to Covidence (https://www.covidence.org/) for eligibility screening by two authors (F.S.H. and E.L.P.). Conflicts were resolved between authors. Initial screening was conducted based on the title and abstract, and further screening was performed based on the full text. Studies were eligible for inclusion in the review if they included (i) preterm infants (defined as gestational age <37 weeks [13]) with NEC (case group; either based on the Bell staging system or by indicating the presence of NEC without specifying the Bell stage) and without NEC (control group) and (ii) examined the gut microbiota using next-generation sequencing-based methods. Studies were eligible to be included if they utilized stool samples, meconium samples or both sample types to characterize the gut microbiota. Studies were excluded if they (i) did not have appropriate case and control groups, (ii) reported on full-term infants or adults only, (iii) were duplicate studies, (iv) were review studies, (v) used non-molecular methods to characterize the gut microbiota or relied only on targeted PCR, (vi) had a low sample size (<5 infants total) or (vii) were performed in animals. Only English language studies were included, with no additional restrictions on study design.

### Data extraction

Data were independently extracted by two authors (F.S.H. and E.L.P.). No conflicts arose during data extraction. The following data were extracted where available: publication year, recruitment period, location, study population, sample type, the total number of collected samples, number of collected samples per participant, sampling time frame, weight and age, inclusion and exclusion criteria, characteristics for matching control and case infants (if applicable), NEC severity including Bell’s stage, sample preservative, DNA extraction method, sequencing platform, 16S rRNA gene variable regions sequenced, primers used, average number of sequencing reads per sample, gut microbiota findings (e.g. relative abundance of microbial communities between case and controls, differential abundance findings, alpha diversity and beta diversity) and limitations (if available).

### Analysis

To comprehensively investigate the gut microbiota composition in preterm infants with and without NEC, a narrative analysis approach was employed. The analysis involved reviewing included studies to explore relevant findings to gut microbiota, prevalent microbial communities, comparisons of alpha and beta diversity and bacterial taxa associated with NEC.

### Risk of bias assessment and certainty of evidence assessment

A risk of bias assessment was conducted by two authors (F.S.H. and E.L.P.) investigating biases in four main domains: (1) sampling bias, (2) comparability bias, (3) data reporting bias and (4) outcome measurement bias. These domains were broken down into subdomains to provide a comprehensive assessment as further defined in Table S1. Studies were defined as low risk if they had an overall score of 0–3 (representing bias in ≤3 subdomains), medium risk if they had an overall score of 4–6 (representing bias in 4–6 subdomains) and high risk if they had an overall score ≥7 (representing bias in 7 or more subdomains). Studies were not excluded based on the risk of bias assessment. The certainty of evidence was assessed using the Grading of Recommendations, Assessment, Development and Evaluation (GRADE) approach outlined by Murad *et al.* for systematic reviews that employ a narrative analysis [14]. The GRADE approach was applied for three microbiota outcomes: alpha diversity, beta diversity and individual taxa and considered five domains: risk of bias, inconsistency, indirectness, imprecision and publication bias. The overall certainty of the evidence was judged as ‘very low’, ‘low’, ‘moderate’ or ‘high’, as outlined by Balshem *et al.* [15].

## Results

### Study selection

**Fig. 1.**
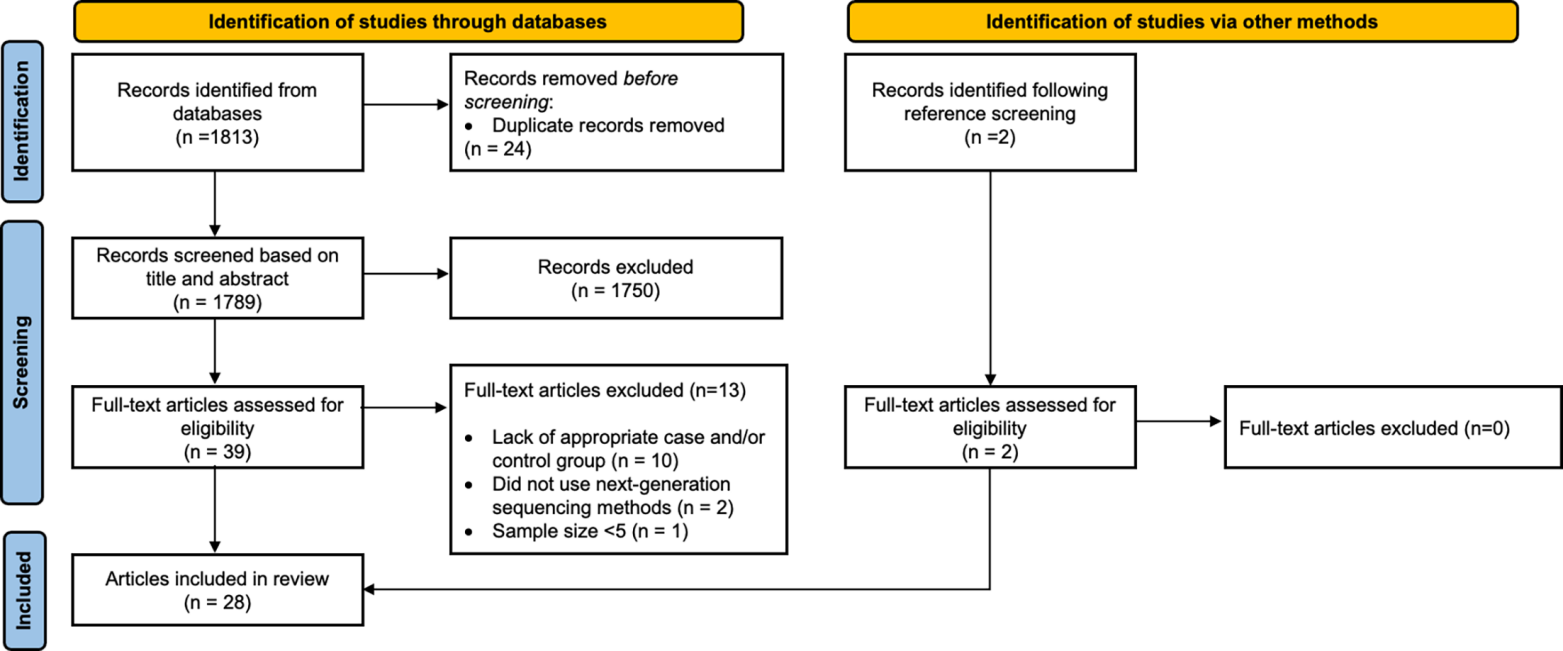
PRISMA flow diagram of the literature search and article selection.

A total of 1,813 relevant records were identified through the database search. Duplicate records (*n*=24) were removed, leaving 1,789 unique records. A total of 1,750 records were identified as irrelevant after title and abstract screening. The remaining records (*n*=39) were assessed for eligibility through full-text screening. Thirteen records were excluded due to lack of appropriate case and control groups (i.e. did not include healthy controls or controls had comorbidities, the case group included infants without NEC, the study did not directly compare infants with and without NEC and the control group included full-term infants; *n*=10), did not use next-generation sequencing methods (i.e. used targeted PCR for specific bacteria; *n*=2) and low sample size (*n*=1). Details of studies excluded following full-text review are provided in Table S2, Supplementary Material 2. An additional two studies were identified as eligible after reviewing the reference list of prior published systematic reviews [11,16]. As a result, 28 eligible studies were included in the review (Fig. 1).

### Study characteristics

Characteristics of included studies are summarized in Table 1, with detailed characteristics provided in Table S3. Fifteen studies were conducted in the USA, three in the UK, France and China and one in Sweden, the Netherlands, Lebanon and Brazil (Table 1).

**Table 1.** Characteristics of included studies

First author*	Year	Recruitment period	Location	No. of participants	Total samples included†	Sample type	Sequencing platform	Key findings
Mshvildadze [17]	2010	nr	USA	12 (6 NEC and/or suspected sepsis, 6 control)	12 (6 NEC, 6 control)	Stool	454 sequencing platform	High abundance of *Citrobacter*-like sequences was identified in three of four infants with NEC, which was absent from controls. The overall microbial pattern was similar between control and cases.
Mai [28]	2011	nr	USA	18 (9 NEC, 9 control)	36	Stool	454 Flex chemistry	High abundance of Proteobacteria and a low abundance of Firmicutes in NEC cases during the week prior to NEC. A specific *γ*-Proteobacteria OTU was present in three of nine NEC cases and was not detected in controls. Lower abundance of Actinobacteria and Bacteroidetes in NEC compared to the control infants. No difference in alpha diversity (Chao-1) between cases and controls.
Normann [18]	2013	June 2009 to June 2010	Sweden	19 (10 NEC, 9 control)	73 (37 NEC, 36 control)	Stool	454 sequencing platform	No significant difference in gut microbiota was observed between infants with NEC and healthy controls. Early NEC onset was non-significantly associated with a high abundance of *Bacillales* and *Enterobacteriaceae*. Low bacterial diversity in both groups.
Claud [21]	2013	nr	USA	10 (5 NEC, 5 control)	56	Stool	454 sequencing platform (subset of samples underwent shotgun sequencing using S-FLX LR70 and XLR70 sequencing chemistry and the Illumina GAIIx platform)	A clear temporal microbiota separation between healthy and controls was detected. A significant difference in overall microbiota composition between NEC and control infants. Samples from infants with NEC diverged from control samples ~3 weeks prior to NEC onset.
Morrow [30]	2013	October 2009 to August 2010	USA	35 (11 NEC, 21 control)	58 (18 NEC, 37 control, 3 non-NEC deaths)	Stool	454 sequencing platform	Low alpha diversity (Chao-1) and lack of Propionibacterium abundance in infants with NEC compared to the healthy controls in samples collected between days 4–9. Late NEC samples (collected days 10–16) were dominated by Proteobacteria, *Enterobacteriaceae*. Firmicutes dysbiosis prior to the NEC occurred earlier than Proteobacteria dysbiosis. Different microbial dysbioses were identified in NEC cases; infants with Firmicutes dysbiosis had earlier onset NEC (7–21 days) than infants with Proteobacteria dysbiosis (19–39 days).
Torrazza [37]	2013	nr	USA	53 (18 NEC, 35 control)	118	Meconium and stool	454 sequencing platform	High abundance of Proteobacteria and Actinobacteria and lower abundance of Bifidobacteria and Bacteroidetes was observed in NEC cases compared to controls, 1 week before NEC diagnosis and in the early stages of NEC. High abundance of *Klebsiella granulomatis*, *K. pneumoniae*, *C. perfringens* and *Staphylococcus epidermidis* in NEC cases close to NEC diagnosis. Alpha diversity was not significantly different between cases and controls. Beta diversity was significantly different between NEC and the control group.
Cassir [41]	2015	February 2009 to March 2013	France	30 (15 NEC, 50 control)	30 (15 NEC, 50 control)	Stool	454 sequencing platform	The detection and relative abundance of *C. butyricum* was higher in infants with NEC compared to control infants. Bacterial diversity (Chao-1 and Shannon diversity) was lower in NEC cases compared to controls.
Sim [35]	2015	January 2010 to December 2012	UK	48 (12 NEC, 36 control)	543 (174 NEC, 369 control)	Stool	454 sequencing platform	High abundance of *Clostridia* (*C. perfringens* identified via culture) and *Klebsiella* in the early onset of NEC. High abundance of *Klebsiella*, *Staphylococcus*, *Enterobacteriaceae*, *Enterococcus* and *Bifidobacterium* in the healthy group. Two groups of NEC infants were identified based on microbial signatures: *Clostridium*-associated NEC cases and *Klebsiella* OTU-associated NEC cases, and the two groups displayed different longitudinal gut microbiota profiles.
McMurtry [29]	2015	2007–2011	USA	95 (21 NEC, 74 control)	95	Stool	454 sequencing platform	A significant decrease in Actinobacteria, *Clostridia*, *Veillonella* and *Streptococcus* abundance in NEC cases compared to controls. Lower bacterial diversity (Chao-1 and Shannon diversity) and lower *Clostridia* abundance and prevalence were reported as NEC severity increased.
Zhou [40]	2015	nr	USA	38 (12 NEC, 26 control)	312	Stool	454 sequencing platform	Early NEC onset was significantly associated with a higher abundance of *Clostridium sensu stricto* and *Staphylococcus*. Late-onset NEC was associated with *Gammaproteobacteria (Escherichia/Shigella* and *Cronobacter*). Variable microbial patterns were observed over NEC progression and as infants grow. *Pasteurella* increased in NEC cases in the second week of life and was absent from controls. The separation between controls and late-onset NEC cases was not as evident as between early-onset cases and controls. Control samples had significantly higher richness over time but no difference in Shannon diversity.
Heida [24]	2016	October 2012 to February 2014	Netherlands	33 (11 NEC, 22 control)	87 (30 NEC, 57 control)	Meconium and stool	Illumina MiSeq	NEC-associated gut microbiota was present within days after birth. High abundance of *C. perfringens* and *B. dorei* in NEC cases, evident in meconium and last two samples before NEC onset. Low abundance of staphylococci in NEC cases, and a higher abundance of lactate-producing bacilli such as staphylococci and streptococci in post-meconium samples, was associated with lower mortality. Low abundance of *Clostridium difficile* in meconium NEC samples compared to controls. Bacterial diversity was not associated with NEC development. *Bacteroidaceae* in the meconium was positively associated with mortality, whereas *C. perfringens* was not.
Stewart [36]	2016	nr	USA	35 (7 NEC, 28 control)	641 (121 NEC, 520 control)	Stool	Illumina MiSeq	NEC samples showed more bacterial variation prior to NEC diagnosis. High abundance of *Bifidobacterium* in the healthy group. Temporal development of Shannon diversity increased in control infants but reduced in NEC samples.
Ward [38]‡	2016	December 2009 to July 2012	USA	144 (27 NEC, 117 control)	405 (including samples from 22 term infants)	Stool	Illumina HiSeq	Early and late NEC cases had similar bacterial diversity compared to infants without NEC. NEC and mortality rate were associated with *Enterobacteriaceae* (Uropathogenic *E. coli*). Lower abundance of *Veillonella* in NEC cases.
Warner [39]	2016	July 2009 to September 2013	USA	122 (28 NEC, 94 control)	2,492	Stool	454 sequencing platform	A higher abundance of *Gammaproteobacteria* and a lower abundance of anaerobic bacteria (*Negativicutes* and combined *Clostridia-Negativicutes*) were associated with NEC onset. Lower bacterial diversity over time in cases compared to controls.
Dobbler [22]	2017	nr	Brazil	40 (11 NEC, 20 control)	132	Meconium and stool	Ion Torrent	NEC was positively associated with a high abundance of *Enterobacteriaceae* (*Ci. koseri* and *K. pneumoniae*) and low abundance of *Lactobacillus*. NEC cases showed lower diversity longitudinally and higher dominance of individual taxa compared to control infants.
Ravi [32]‡	2017	nr	USA	62 (23 NEC, 39 control)	160 (63 NEC, 97 control)	Stool	Illumina MiSeq	No significant difference in alpha diversity between infants with and without NEC was reported. Beta diversity was significantly different between NEC and non-NEC samples. A significantly higher abundance of *Enterobacteriaceae* in samples from infants with NEC. One *Enterobacteriaceae* OTU (OTU2) was significantly correlated and associated with NEC. OTU2 had the highest identity to HG428755, an enteropathogenic *E. coli*.
Rozé [33]	2017	2011	France	72 (15 NEC, 57 control)	nr	Stool	454 sequencing platform	Association of *Clostridium sensu stricto* (and specific *Clostridia* OTUs, *C. neonatale* and *C. butyricum*) and *Gammaproteobacteria* (*Enterobacteriaceae*) with NEC. Lower proportions of *Klebsiella* and *Citrobacter* in NEC cases.
Wandro [19]	2018	2011–2014	USA	24 (3 NEC, 21 control)	45	Stool	Illumina MiSeq	No association was observed between NEC and gut microbiota.
Feng [42]	2019	May 2016 to February 2018	China	32 (16 NEC, 16 control)	32 (16 NEC, 16 control)	Stool	Illumina MiSeq	No difference between cases and controls at the phylum level. The abundance of *Propionibacteriales* was higher in infants with NEC compared to controls, and the abundance of *Lactobacillus*, *Phascolarctobacterium* and *Streptococcus salivarius* was higher in control infants compared to NEC cases. No difference in richness or beta diversity between cases and controls.
Gopalakrishna [23]	2019	nr	USA	23 (10 NEC, 13 control)	98 (39 NEC, 59 control)	Stool	Illumina MiSeq	A modest enrichment of *Enterobacteriaceae* (non-significant) and a reduction in Gram-positive anaerobes (*Lachnospiraceae*) in NEC cases compared to healthy preterm infants.
Itani [25]	2019	January 2013 to March 2015	Lebanon	22 (11 NEC, 11 control)	nr	Stool	Illumina MiSeq	Higher abundance of Firmicutes and Proteobacteria in NEC and control samples collected at the first time point of sampling. Higher numbers of *Enterococcaceae*, *Streptococcaceae* and *Lactobacillaceae* in controls at the first two time points. High abundance of *Staphylococcaceae* before and during NEC onset. Lower alpha diversity in infants with NEC at all time points. No difference in *Enterobacteriaceae* between infants with and without NEC.
Liu [27]	2019	July 2013 to December 2014	China	21 (4 NEC, 17 control)	148 (45 NEC, 103 control)	Stool	Illumina MiSeq	Highly diversified microbiota structure was detected in all preterm infants shortly after birth. Higher abundance of *Bacillus* and *Solibacillus* in NEC cases preceding the disease. A rapid increase in *Enterococcus*, *Staphylococcus* and *Streptococcus* from pre-onset to early disease was only observed in infants with NEC, with dominance of *Enterococcus*, *Streptococcus* and *Peptoclostridium* in NEC cases during disease phases, and decreased alpha diversity [observed species richness (Sobs) and Shannon diversity] in all cases and controls over time through to disease remission. Beta diversity of the NEC increasingly diverged as the diseases got worse but then converged once the conditions improved. Shannon’s diversity levels increased back to early pre-onset levels post-NEC.
Olm [31]‡	2019	Various cohorts between 2011 and 2015	USA	160 (34 NEC, 126 control)	1,163 (282 NEC, 881 control)	Stool	Illumina HiSeq	Higher abundance of *Klebsiella*, bacteria encoding fimbriae and replication of *Enterobacteriaceae* prior to NEC diagnosis. Lower abundance of Firmicutes and higher abundances of *Enterobacteriaceae* (but not significant) in NEC compared to the control. *K. pneumoniae* strain 242_2 associated with NEC, present in 52% of NEC vs 20% of controls. High plasmid abundance was observed in NEC infants. Specifically, higher abundance of *K. pneumoniae* and *C. perfringens* plasmids prior to NEC.
Brehin [20]	2020	nr	France	27 (11 NEC, 21 control)	80	Stool	Illumina MiSeq	NEC was associated with a higher abundance of *Streptococcus* and bacteria from the *Micrococcales* order in the second 10 days of life and a higher abundance of *Staphylococcus* and *Streptococcus* in the third 10 days of life. An increase in *Raoultella* species in NEC-1 gut microbiota compared to controls was observed in the second month of life. The microbial diversity was lower in infants with NEC-1 compared to controls in the first 10 days of life, but no difference in microbial diversity was observed between NEC-1 and controls at either third 10 days of life or second month.
Lindberg [26]	2020	September 2013 to September 2015	USA	10 (5 NEC, 5 control)	29 (15 NEC, 14 control)	Stool	Illumina MiSeq	NEC was associated with a higher abundance of Proteobacteria specifically *Gammaproteobacteria* (*Enterobacteriaceae* and *Trabulsiella*). Higher abundance of Firmicutes was observed in healthy preterm infants. Diversity was not associated with NEC after adjusting for the day of life and antibiotic exposure.
Masi [10]‡	2021	nr	UK	48 (14 NEC, 34 control)	644	Stool	HiSeq X Ten	Lower abundance of Actinobacteria (*Bi. longum*) and a higher abundance of Proteobacteria (*En. cloacae*) in NEC samples compared to the control group. Higher bacterial richness in the control group, but based on the Shannon diversity, there was no significant difference between gut microbiota in NEC and the control groups. The relative abundance of *Escherichia* unclassified was higher in NEC cases.
Fu [2]	2021	February 2018 to April 2019	China	30 (15 NEC, 15 control)	nr	Stool	Illumina NovaSeqPE250	Higher alpha diversity in NEC compared to the healthy controls, higher microbial similarity at the onset of NEC in controls compared to the NEC. Higher abundance of Bacteroides and Actinobacteria, *Rhizobiales*, *Dysgonomonas*, *Ochrobactrum*, *Ralstonia*, *Pelomonas* and *Acinetobacter* in NEC infants. Higher abundance of waterborne bacteria in NEC.
Shaw [34]‡	2021	January 2011 to December 2012	UK	24 (12 NEC, 12 control)	22 (11 NEC, 11 control)	Stool	Illumina NextSeq 500	No significant difference in bacterial abundance was found between control and NEC samples. However, two groups of infants with NEC were identified. One with a high abundance of bacterial species expressing LPS content and one with low levels of CpG DNA content.

*Additional study characteristics are presented in Table S3.

†Refers to the total number of samples analysed in the study. This value may be larger than the number of participants if participants provided >1 sample.

‡Study used metagenomic sequencing.

nr, Not reported.

The number of participants varied across the studies, ranging from 10 to 160 in total, and most studies were longitudinal in design or included >1 sample per participant. Most studies utilized stool samples (*n*=25) or a combination of stool and meconium samples (*n*=3). DNA extraction was predominantly performed using the QIAamp DNA Mini Stool Kit (*n*=8) and MoBio PowerSoil Kit (*n*=6). Twenty-three studies used 16S rRNA gene sequencing to characterize the microbiota, four used shotgun metagenomic sequencing and one used both 16S rRNA gene sequencing and shotgun metagenomics. The V3 and V4 regions (*n*=11) were the most common variable regions studied. In 23 studies, cases and controls were matched for potential confounding factors, with gestational age and birth weight being the primary variables used for matching.

### Bacterial taxa commonly present in infants without NEC

The dominant phyla reported in healthy preterm infants were Proteobacteria, Firmicutes, Actinobacteria and Bacteroidetes. Within these phyla, specific bacterial classes, families and genera were consistently identified in infants without NEC, including *Enterobacteriaceae*, *Staphylococcus*, *Klebsiella*, *Bifidobacterium* and *Enterococcus*.

### Bacterial taxa commonly present in infants with NEC

Dominant taxa present in infants with NEC largely overlapped with those detected in healthy infants. Proteobacteria was the most commonly detected phylum in NEC cases, with high abundance reported in several studies. Actinobacteria and Firmicutes were identified as abundant in individuals with NEC, although to a lesser extent. At the class, family and genus levels, *Enterobacteriaceae*, *Staphylococcus*, *Klebsiella, Enterococcus* and *Streptococcus* were also commonly reported in infants with NEC. *Bifidobacterium* was not commonly recovered from infants with NEC.

### Comparison of gut microbiota composition between NEC and control groups

Based on various analytical approaches to microbial analysis (including richness and evenness, taxonomic classification and differential abundance analysis), three studies, each using 16S rRNA gene sequencing to characterize the microbiota, reported no difference in gut microbiota composition between NEC and control groups [17,19]. Importantly, 25 studies reported a difference in gut microbiota composition between NEC and control infants using one or more analytical approaches (20 used 16S rRNA gene sequencing, 4 used shotgun metagenomics and 1 used both sequencing approaches) [2,10, 20,42].

### Microbial diversity in control and NEC groups

Twenty-four studies compared alpha diversity between NEC cases and healthy infants with variable results. The most used metrics to measure alpha diversity included the Shannon diversity index (*n*=17), Chao-1 index (*n*=9) and Simpson diversity index (*n*=4), and some studies used more than one method to measure alpha diversity (Table S3). In total, 11 studies reported lower alpha diversity (using one or more diversity metric) in the NEC group compared to the control group [10,20, 22, 23, 25, 29, 30, 36, 39,41]; 1 study reported higher diversity in the NEC group [2] and 12 studies found no statistically significant difference in alpha diversity between the two groups [18,19, 24, 26,28, 31, 32, 35, 37, 38, 42] (Table 2). Because different diversity metrics may yield contradictory results, we reviewed alpha diversity findings generated using the two most used metrics: the Shannon diversity index and Chao-1 index. Of the 17 studies that applied the Shannon diversity index, 8 reported a lower alpha diversity in the NEC group compared to the control group, and 9 reported no difference. Similarly, of the nine studies that applied the Chao-1 index to estimate richness, five reported lower richness in NEC cases compared to controls, and four reported no difference between the groups.

**Table 2.** Microbial diversity findings of included studies

Analysis	Study finding	No. of studies reporting the result
Alpha diversity (*n*=24 studies)*	Lower alpha diversity in NEC group compared to control group using one or more diversity metric	11
	Higher alpha diversity in NEC group compared to control group using one or more diversity metric	1
	No difference between NEC and control groups	12
Beta diversity (*n*=19 studies)†	Distinct clustering of samples collected from infants with NEC compared to control infants at one or more time points	9
	No distinct clustering of samples collected from infants with NEC and control infants	10

*Alpha diversity not reported or not compared between NEC and control groups in *n*=4 studies.

†Beta diversity not reported or not compared between NEC and control groups in *n*=9 studies.

Importantly, 19 studies compared beta diversity between NEC cases and healthy controls, with weighted and unweighted UniFrac distances (*n*=5 weighted, *n*=6 unweighted and *n*=2 weighted/unweighted not specified], and Bray–Curtis dissimilarity (*n*=3) being the most common metrics used to assess beta diversity (Table S3). Similar to alpha diversity, beta diversity findings across studies were heterogenous. Case and control groups tended to cluster separately in nine studies [2,20, 21, 25,27, 32, 37, 40], indicating a difference in the global microbiota composition between infants with and without NEC (Table 2). In contrast, ten studies showed no obvious distinct clustering of NEC and control groups or reported no significant difference in beta diversity metrics [10,17,19, 24, 28]. Of the six studies that applied weighted UniFrac, two reported a distinct clustering of (or a difference between) NEC and control groups, and four reported no difference. Interestingly, one longitudinal analysis that used the Bray–Curtis dissimilarity metric reported a significant difference in global microbiota composition at 2 weeks of life between healthy controls and infants who had early-onset NEC (≤22 days at diagnosis), but not at 3 weeks of life. Furthermore, no significant difference was observed in global microbiota composition between healthy infants and infants who had late-onset NEC (>22 days at diagnosis) [40].

### The association between specific taxa and NEC

The assessment of bacterial associations with NEC generally yielded contrasting findings across studies, but there were some commonalities (Table 3). Of note, two studies reported a positive association between NEC and increased abundance of Proteobacteria (specifically *Gammaproteobacteria*) [26,28]. Further, 11 studies reported a positive association between NEC and organisms from the *Enterobacteriaceae* family, including *Enterobacter*, *Escherichia*, *Klebsiella pneumoniae* and *Citrobacter koseri* [10,21,23, 30].

**Table 3.** Bacterial taxa positively associated with NEC

Taxon	No. of studies reporting an association between NEC and taxon
Proteobacteria	2
*Enterobacteriaceae*: including *Enterobacter*, *Escherichia*, *Ci. koseri*, *K. pneumoniae*, *Klebsiella granulomatis* and *En. cloacae*	11
*Clostridium* spp.: including *C. neonatale*, *C. perfringens* and *C. butyricum*	8

In addition, eight studies identified an association between *Clostridium* and NEC [24,29, 33, 35, 37, 39,41]. In detail, one study reported a positive association between NEC development and high abundance of *Clostridium perfringens* and *Bacteroides dorei* in meconium samples collected prior to NEC onset [24]. A second showed two distinct microbial signatures in samples collected from infants who later developed NEC, one of which was an overabundance of a clostridial operational taxonomic unit (OTU) (confirmed by culture to likely be *C. perfringens* type A) and the second an overabundance of a *Klebsiella* OTU [35]. Cassir *et al.* found *Clostridium butyricum* was more prevalent and more abundant in infants with NEC compared to infants without NEC, using samples collected on the day of symptom onset [41], and high colonization of *Clostridium neonatale* and *Staphylococcus aureus* at the time NEC was reported by Rozé *et al.* [33]. In another study [40], early-onset NEC was associated with increased abundance of *Clostridium sensu stricto*, whereas infants with late-onset NEC had increased abundance of *Escherichia*/*Shigella* (and *Cronobacter*) compared to controls in the lead up to disease onset. These data suggest that the aetiology of NEC (or gut microbiota composition) may differ by age of NEC onset [30]*.*

### Insights from metagenomic analyses

Five articles used metagenomic sequencing to investigate the relationship between NEC and gut microbiota composition [10,31, 32, 34, 38] (Table 1). Ward *et al.* identified three functionally distinct clades of *Escherichia coli* strains present in study participants, two of which included samples from infants who later developed NEC [38]. Further, the presence of uropathogenic *E. coli* strains (identified using MLST assignment) was associated with increased odds of both NEC and NEC-associated death. Using 16S rRNA gene sequencing, Ravi *et al.* identified an OTU that was enriched in infants with NEC, which, using metagenomics, was identified as closely related to HG428755, an enteropathogenic *E. coli* [32]. Authors also reported high levels of accessory genes related to virulence factors and antibiotic resistance in infants with NEC; however, the metagenomic analysis was limited to only five infants [32].

Olm *et al.* reported enrichment of *K. pneumoniae* in infants with NEC and found that increased bacterial replication rates, presence of secondary metabolite clusters (sactipeptides, bacteriocins and butyrolactones), presence of *Klebsiella* and presence of bacterial fimbriae encoding gene clusters were important biomarkers increased in pre-NEC samples compared to controls [31]. Shaw *et al.* described two microbial communities in infants with NEC, one characterized by high abundance of bacterial species expressing LPS content and one with low levels of CpG DNA content [34]. The authors hypothesized that these community patterns support immunological studies that suggest a role for Toll-like receptor (TLR) mediated pathways in NEC development. Finally, Masi *et al.* performed an integrated analysis of human milk oligosaccharide (HMO) profiles and metagenomic profiles [10]. Authors reported decreased concentration of a single HMO (disialyllacto-*N*-tetraose) in infants with NEC, which was also associated with reduced progression towards a gut microbiota abundant in *Bifidobacterium* spp. Of note, increased abundance of *Enterobacter cloacae* and decreased abundance of *Bifidobacterium longum* were both positively associated with NEC in this study.

### Risk of bias assessment and certainty of evidence assessment

Of the included articles, 3 had a high risk of bias [17,19, 21], 15 had a medium risk [10,18, 22,25, 27, 28, 31, 33] and 10 had a low risk of bias [2,20, 26, 29, 30, 32, 37,40] (Table S1). No study had a low risk of bias across all domains. The subdomains in which studies showed the highest risk of bias were the absence of experimental controls, including DNA extraction and PCR controls (D13, *n*=25 studies), and small sample size (D1, *n*=21 studies included fewer than 50 infants).

Table S4 presents the certainty of evidence for an association between NEC and the three microbiota outcomes examined (alpha diversity, beta diversity and abundance and/or prevalence of taxa) using the GRADE approach. The overall certainty of evidence was graded as ‘low’ for each outcome due to issues of inconsistency (heterogeneity between study findings) and imprecision (most studies had small sample sizes of <50 participants).

## Discussion

This systematic review aimed to determine differences in gut microbiota composition between preterm infants with and without NEC, with a focus on studies using next-generation sequencing methods. While 25 of 28 studies included in the review reported a difference using one or more analytical method, no specific microbial signature or pathogen was consistently associated with NEC across all studies. Importantly, most studies in this review assessed a small number of NEC cases, and the overall certainty of evidence was deemed to be ‘low’.

Lower alpha diversity was reported in the NEC group compared to the control group in 11 studies, suggesting an association between NEC and a low diversity gut microbiota, which might be due to factors such as increased antibiotic exposure and more controlled feeding regimen in infants with NEC compared to healthy controls [43]. Lower alpha diversity has also been identified in other gut microbiota-related diseases, such as inflammatory bowel disease, irritable bowel syndrome and coeliac disease [44,46]. While the precise mechanisms aren't fully understood, it is hypothesized that lower gut microbiota diversity may promote the growth of pathogenic bacteria, which may contribute to gut microbiota-associated diseases. Additionally, a less diverse microbiota may struggle to adapt to environmental changes like feeding adjustments or antibiotic use [4,11, 39]. However, 12 studies reported no association between NEC and alpha diversity. Beta diversity findings were also heterogeneous. Inconsistencies in diversity findings may be related to numerous factors, including differences in timing of sampling relative to time of birth and/or time of NEC diagnosis, differences in gestational age, differences in sequencing methodology and/or differences in analytical metrics used to assess bacterial diversity. Regardless, heterogeneous diversity findings were observed even when single diversity metrics were examined. In their re-analysis of 16S rRNA gene data from eight studies, Pammi *et al.* found no difference in alpha or beta diversity metrics between infants with and without NEC but noted a non-significant finding of lower species richness in NEC cases compared to controls when gestational age was controlled for [11]. Further research is needed to understand the relationship between NEC and gut microbiome diversity.

Our review identified that the class *Gammaproteobacteria* was commonly recovered from the gut of preterm infants with NEC [22,26, 33, 39, 40]. Further investigation on lower taxonomic ranks showed that this class includes an important bacterial family (*Enterobacteriaceae*) which was abundant in the NEC groups in several studies [18,19, 21, 23, 29,32, 34, 38] and positively associated with NEC development in multiple studies [10,21,23, 30]. According to previous findings, colonization of the gut with *Enterobacteriaceae* can promote intestinal inflammation, disrupt the intestinal barrier and induce oxidative stress, which can contribute to the development of NEC [11,39]. Furthermore, the importance of *Enterobacteriaceae* in preterm infants may also lie in their ability to modulate the immune system through the activation of TLRs [47,48].

Importantly, the specific bacterial taxa identified in this review, such as *Enterobacteriaceae* and *Clostridium* spp., underscore that these micro-organisms may have a role in the pathogenesis of NEC and could be explored as a potential microbial signature for NEC risk assessment. One might expect to detect *Clostridium* spp. from the gut microbiota of infants with advanced NEC, particularly in the presence of necrosed tissue [49]. Therefore, it was interesting to note that several studies reported high abundance of *Clostridium* spp. prior to NEC onset or at the time of NEC diagnosis. This may suggest that *Clostridium* spp. play a role in NEC development and further highlight the importance of characterizing the evolution of the gut microbiota preceding, during and after NEC onset. Further research should evaluate the contribution of these taxa to NEC and explore the potential of microbiota-targeted interventions for NEC.

This review presents the most extensive collection of studies on gut microbiota composition between healthy preterm infants and preterm infants with NEC to date [11,16]; however, there are limitations. Almost all included studies primarily focused on bacterial organisms. As a result, our review was limited to examining the association between NEC and the bacterial composition of the gut. Other micro-organisms such as viruses and bacteriophages and fungi may have a role in NEC development [1], highlighting an important area for future research. Furthermore, altered microbial functions may underlie NEC pathogenicity, and studies examining changes in the proteome and/or metabolome associated with NEC are likely to provide important insights into the aetiology of NEC [16].

Our review specifically focused on studies that applied next-generation sequencing-based methods, which can identify rare bacterial taxa and difficult to culture bacteria, thereby providing a comprehensive picture of the gut microbiota in preterm infants. However, most studies used 16S rRNA gene sequencing, which has limited resolution beyond the genus level, and several only characterized the gut microbiome at higher taxonomic levels (i.e. family or phylum). This not only makes it difficult to compare findings across studies but may also result in an overestimation of the association between individual bacterial taxa and NEC [38]. Studies utilizing metagenomic sequencing reported differences in the functional potential of the microbiota, as well as taxonomic composition, of infants with and without NEC and in the lead-up to NEC, highlighting the complex nature of NEC pathogenesis.

In addition to sequencing approaches, it is important to note that methodological variability related to study design, inclusion/exclusion criteria, NEC diagnostic criteria, sample type and sample collection method may influence microbiota findings and make it difficult to directly compare studies. For example, the use of preservatives in stool collection tubes (for example, RNA-Later, PBS) and sample storage temperatures differed between studies and can impact microbiome composition. Additionally, molecular-based methods are associated with technical challenges in DNA extraction, sequencing platforms and data analysis pipelines which can potentially introduce biases towards specific taxa. Finally, factors like birth mode, feeding practices, antibiotic usage, sample collection timing relative to NEC diagnosis and age at NEC diagnosis can influence the gut microbiota composition [11,50]. A limitation of our review is that we were unable to account for these variables in a systematic manner as this data were not consistently reported for all studies.

While some methodological variation is expected, establishing consistency in data collection, analysis and reporting for future studies would greatly benefit our understanding of the association between NEC and gut microbiota dysbiosis. This may include accurate descriptions and reporting of NEC diagnosis and severity, sample collection timing with respect to birth (and/or gestational age) and NEC onset, as well as feeding practices and antibiotic use. Furthermore, the use of standardized reporting guidelines, such as the STORMS (Strengthening The Organization and Reporting of Microbiome Studies) checklist, is recommended to ensure transparent and reproducible research and to facilitate the comparative analysis of studies [51].

Overall, future studies with larger sample sizes, particularly those with longitudinally collected samples, are needed to determine the gut microbiota composition and its functions over time and improve our understanding of the impact of gut microbiota on preterm infant health. Additionally, most studies included in the review were from the USA and few were from resource-limited countries, highlighting an important area for future study.

## Conclusion

In conclusion, most of the included studies reported a difference in the gut microbiota composition of infants with and without NEC, supporting an association between NEC and gut microbiota dysbiosis. However, the reported differences were heterogeneous, and the certainty of the evidence was low. It is important to acknowledge that a single factor or microbial community cannot fully explain the pathogenicity of this disease. Instead, it is likely that different microbial communities and several host factors contribute to the development of NEC, and this should be considered in future studies.

## Supplementary material

10.1099/acmi.0.001077.v3 Supplementary Material 1.

10.1099/acmi.0.001077.v3 Supplementary Material 2.

## Abbreviations

GRADE: Grading of Recommendations, Assessment, Development and Evaluation
HMO: human milk oligosaccharide
NEC: necrotizing enterocolitis
OTU: operational taxonomic unit
PRISMA: Preferred Reporting Items for Systematic Reviews and Meta-analyses
TLR: Toll-like receptor
