# Supplementary material for: Gut microbiota composition in preterm infants with and without necrotizing enterocolitis: a systematic review and narrative synthesis
Source: Access Microbiol. 2025 Dec 19;7(12):001077.v3. doi: 10.1099/acmi.0.001077.v3 (PMC12720982; doi:10.1099/acmi.0.001077.v3)
Supplement: Supplementary Material 1. [file acmi-7-01077-s001.pdf]

PRISMA Checklist

From: Page MJ, McKenzie JE, Bossuyt PM, Boutron I, Hoffmann TC, Mulrow CD, et al. The PRISMA 2020 statement: an updated guideline for reporting systematic reviews. BMJ 2021;372:n71. doi: 10.1136/bmj.n71
For more information, visit: http://www.prisma-statement.org/

Section and Topic	Item #	Checklist item	Location where item is reported
**TITLE**			
Title	1	Identify the report as a systematic review.	Page 1
**ABSTRACT**			
Abstract	2	See the PRISMA 2020 for Abstracts checklist.	Page 3
**INTRODUCTION**			
Rationale	3	Describe the rationale for the review in the context of existing knowledge.	Page 4 - 5
Objectives	4	Provide an explicit statement of the objective(s) or question(s) the review addresses.	Page 5
**METHODS**			
Eligibility criteria	5	Specify the inclusion and exclusion criteria for the review and how studies were grouped for the syntheses.	Page 5
Information sources	6	Specify all databases, registers, websites, organisations, reference lists and other sources searched or consulted to identify studies. Specify the date when each source was last searched or consulted.	Page 5
Search strategy	7	Present the full search strategies for all databases, registers and websites, including any filters and limits used.	Page 5
Selection process	8	Specify the methods used to decide whether a study met the inclusion criteria of the review, including how many reviewers screened each record and each report retrieved, whether they worked independently, and if applicable, details of automation tools used in the process.	Page 5
Data collection process	9	Specify the methods used to collect data from reports, including how many reviewers collected data from each report, whether they worked independently, any processes for obtaining or confirming data from study investigators, and if applicable, details of automation tools used in the process.	Page 6
Data items	10a	List and define all outcomes for which data were sought. Specify whether all results that were compatible with each outcome domain in each study were sought (e.g. for all measures, time points, analyses), and if not, the methods used to decide which results to collect.	Page 6
	10b	List and define all other variables for which data were sought (e.g. participant and intervention characteristics, funding sources). Describe any assumptions made about any missing or unclear information.	Page 6
Study risk of bias assessment	11	Specify the methods used to assess risk of bias in the included studies, including details of the tool(s) used, how many reviewers assessed each study and whether they worked independently, and if applicable, details of automation tools used in the process.	Page 6-7
Effect measures	12	Specify for each outcome the effect measure(s) (e.g. risk ratio, mean difference) used in the synthesis or presentation of results.	NA
Synthesis methods	13a	Describe the processes used to decide which studies were eligible for each synthesis (e.g. tabulating the study intervention characteristics and comparing against the planned groups for each synthesis (item #5)).	Page 6
	13b	Describe any methods required to prepare the data for presentation or synthesis, such as handling of missing summary statistics, or data conversions.	NA
	13c	Describe any methods used to tabulate or visually display results of individual studies and syntheses.	NA
	13d	Describe any methods used to synthesize results and provide a rationale for the choice(s). If meta-analysis was performed, describe the model(s), method(s) to identify the presence and extent of statistical heterogeneity, and software package(s) used.	Page 6

Section and Topic	Item #	Checklist item	Location where item is reported
	13e	Describe any methods used to explore possible causes of heterogeneity among study results (e.g. subgroup analysis, meta-regression).	NA
	13f	Describe any sensitivity analyses conducted to assess robustness of the synthesized results.	NA
Reporting bias assessment	14	Describe any methods used to assess risk of bias due to missing results in a synthesis (arising from reporting biases).	NA
Certainty assessment	15	Describe any methods used to assess certainty (or confidence) in the body of evidence for an outcome.	Page 7
**RESULTS**			
Study selection	16a	Describe the results of the search and selection process, from the number of records identified in the search to the number of studies included in the review, ideally using a flow diagram.	Page 7
	16b	Cite studies that might appear to meet the inclusion criteria, but which were excluded, and explain why they were excluded.	Page 7 (and Table S2)
Study characteristics	17	Cite each included study and present its characteristics.	Page 8, Table 1, Table S3
Risk of bias in studies	18	Present assessments of risk of bias for each included study.	Page 13, Table S1
Results of individual studies	19	For all outcomes, present, for each study: (a) summary statistics for each group (where appropriate) and (b) an effect estimate and its precision (e.g. confidence/credible interval), ideally using structured tables or plots.	NA
Results of syntheses	20a	For each synthesis, briefly summarise the characteristics and risk of bias among contributing studies.	Page 8-13
	20b	Present results of all statistical syntheses conducted. If meta-analysis was done, present for each the summary estimate and its precision (e.g. confidence/credible interval) and measures of statistical heterogeneity. If comparing groups, describe the direction of the effect.	NA
	20c	Present results of all investigations of possible causes of heterogeneity among study results.	NA
	20d	Present results of all sensitivity analyses conducted to assess the robustness of the synthesized results.	NA
Reporting biases	21	Present assessments of risk of bias due to missing results (arising from reporting biases) for each synthesis assessed.	NA
Certainty of evidence	22	Present assessments of certainty (or confidence) in the body of evidence for each outcome assessed.	Page 13
**DISCUSSION**			
Discussion	23a	Provide a general interpretation of the results in the context of other evidence.	Page 14-16
	23b	Discuss any limitations of the evidence included in the review.	Page 16-17
	23c	Discuss any limitations of the review processes used.	Page 16-17
	23d	Discuss implications of the results for practice, policy, and future research.	Page 17-18
**OTHER INFORMATION**			
Registration and protocol	24a	Provide registration information for the review, including register name and registration number, or state that the review was not registered.	Page 5
	24b	Indicate where the review protocol can be accessed, or state that a protocol was not prepared.	Page 5
	24c	Describe and explain any amendments to information provided at registration or in the protocol.	NA

Section and Topic	Item #	Checklist item	Location where item is reported
Support	25	Describe sources of financial or non-financial support for the review, and the role of the funders or sponsors in the review.	Page 35
Competing interests	26	Declare any competing interests of review authors.	Page 35
Availability of data, code and other materials	27	Report which of the following are publicly available and where they can be found: template data collection forms; data extracted from included studies; data used for all analyses; analytic code; any other materials used in the review.	NA

Table S1 – Risk of bias assessment[a]

Study	Bias in sampling			Bias in comparability				Bias in data reporting					Bias in outcome measurement	Overall Score	Bias Risk[b]
	D1	D2	D3	D4	D5	D6	D7	D8	D9	D10	D11	D12	D13		
Mshvildadze, 2010	1	1	1	0	1	0	0	1	1	1	0	0	1	8	High risk
Mai, 2011	1	1	0	0	0	0	0	1	0	0	0	0	1	4	Medium risk
Normann, 2013	1	1	0	1	0	0	0	1	0	0	1	0	1	6	Medium risk
Claud, 2013	1	1	0	1	1	0	0	0	0	1	1	0	1	7	High risk
Morrow, 2013	1	0	0	0	0	0	1	0	0	0	0	0	1	3	Low risk
Torrazza, 2013	0	0	0	0	0	0	0	1	0	0	0	0	1	2	Low risk
Cassir, 2015	1	0	1	1	1	0	0	0	0	1	0	0	1	6	Medium risk
Sim, 2015	1	0	0	1	0	0	0	1	0	1	0	0	1	5	Medium risk
McMurtry, 2015	0	0	1	1	0	0	0	0	0	0	0	0	1	3	Low risk
Zhou, 2015	1	1	0	1	0	0	0	0	0	0	0	0	0	3	Low risk
Heida, 2016	1	1	0	0	0	0	0	1	0	0	0	0	1	4	Medium risk
Stewart, 2016	1	1	0	1	0	0	0	0	0	0	1	0	1	5	Medium risk
Ward, 2016	0	0	0	0	0	0	NR	0	0	1	0	0	1	2	Low risk
Warner, 2016	0	0	0	0	0	0	0	0	0	1	0	0	0	1	Low risk
Dobbler, 2017	1	1	0	0	0	0	0	0	0	1	0	0	1	4	Medium risk
Ravi, 2017	0	0	0	1	0	0	1	0	0	0	0	0	1	3	Low risk
Rozé, 2017	0	0	0	0	0	0	0	1	1	1	0	0	1	4	Medium risk
Wandro, 2018	1	1	0	1	1	0	1	1	0	0	1	0	1	8	High risk
Feng, 2019	1	1	1	0	1	0	0	0	0	0	0	0	1	5	Medium risk
Gopalakrishna, 2019	0	1	0	1	1	0	0	0	0	1	0	0	1	5	Medium risk
Itani, 2019	1	NR	0	1	1	NR	NR	0	0	0	0	0	1	4	Medium risk
Liu, 2019	1	1	0	0	0	0	1	0	0	0	0	0	1	4	Medium risk
Olm, 2019	0	1	0	1	1	0	0	1	0	0	0	0	1	5	Medium risk
Brehin, 2020	1	1	0	0	0	0	0	0	0	0	0	0	1	3	Low risk
Lindberg, 2020	1	1	0	0	0	0	0	0	0	0	0	0	1	3	Low risk
Masi, 2021	1	1	0	1	1	0	0	0	0	0	0	0	1	5	Medium risk

Fu, 2021	1	1	0	0	0	0	0	0	0	0	0	0	1	3	Low risk
Shaw, 2021	1	0	1	0	0	0	0	1	1	1	1	0	0	6	Medium risk

NR; not reported;

[a] Definitions of risk of bias subdomains are provided below:
D1 - Sample size: adequate, i.e. >49 (0), low (1)
D2 - Recruitment: Multi-center recruitment (0), single-center recruitment (1)
D3 - Longitudinal samples: Yes (0), No (1)
D4 - Inclusion and exclusion criteria: reported (0), Not defined (1)
D5 - NEC diagnostic method: Reported (0), Not reported (1)
D6 - Control and NEC were from the same clinical setting and same time frame: Yes (0), No (1)
D7 - Confounding factors: Matched or adjustment in analysis for confounders (0), Not-matched (1)
D8 - Any microbial composition reported as "Other": No (0), Yes (1)
D9 - Alpha diversity: Assessed (0), Not assessed (1)
D10 - Beta diversity: Assessed (0), Not assessed (1)
D11 - Differential abundance analyses: Assessed (0), Not assessed (1)
D12 - Sample type: Reported (0), Not reported (1)
D13 - Experimental controls (e.g. negative controls including DNA extraction and PCR controls, and/or positive controls including community standard controls): Included (0), Not included (1)

[b] Studies were defined as low risk if they had an overall score of 0-3 (representing bias in 0-3 subdomains), medium risk if they had an overall score of 4-6 (representing bias in 4-6 subdomains), and high risk if they had an overall score ≥7 (representing bias in 7 or more subdomains).

Table S2 – Details of studies excluded following full text review

Reference	Primary reason for exclusion
Barron, L. K. et al. Independence of Gut Bacterial Content and Neonatal Necrotizing Enterocolitis Severity. *J Pediatr Surg* **52**, 993-998 (2017).	Lack of appropriate case and/or control group
Brown, C. T. et al. Hospitalized Premature Infants Are Colonized by Related Bacterial Strains with Distinct Proteomic Profiles. *mBio* **9** (2018)	Lack of appropriate case and/or control group
Campeotto, F. et al. Faecal Calprotectin and Gut Microbiota Do Not Predict Enteropathy in Very Preterm Infants. *Acta Paediatr* **110**, 109-116 (2021).	Lack of appropriate case and/or control group
Hong, L. et al. Impacts of Enriched Human Milk Cells on Fecal Metabolome and Gut Microbiome of Premature Infants with Stage I Necrotizing Enterocolitis: A Pilot Study. *Mol Nutr Food Res* **66**, e2100342 (2022).	Lack of appropriate case and/or control group
Hui, Y. et al. The Effect of Early Probiotic Exposure on the Preterm Infant Gut Microbiome Development. *Gut Microbes* **13**, 1951113 (2021).	Lack of appropriate case and/or control group
Kaelin, E. A. et al. Longitudinal Gut Virome Analysis Identifies Specific Viral Signatures That Precede Necrotizing Enterocolitis Onset in Preterm Infants. *Nat Microbiol* **7**, 653-662 (2022).	Did not use next-generation sequencing methods
Leach, S. T. et al. Multiple Opportunistic Pathogens, but Not Pre-Existing Inflammation, May Be Associated with Necrotizing Enterocolitis. *Dig Dis Sci* **60**, 3728-3734 (2015).	Lack of appropriate case and/or control group
Romano-Keeler, J. et al. Distinct Mucosal Microbial Communities in Infants with Surgical Necrotizing Enterocolitis Correlate with Age and Antibiotic Exposure. *PLoS One* **13**, e0206366 (2018).	Lack of appropriate case and/or control group
Sher, Y. et al. Combined Analysis of Microbial Metagenomic and Metatranscriptomic Sequencing Data to Assess in Situ Physiological Conditions in the Premature Infant Gut. *PLoS One* **15**, e0229537 (2020).	Sample size <5
Ward, D. V. et al. Metagenomic Sequencing with Strain-Level Resolution Implicates Uropathogenic E. Coli in Necrotizing Enterocolitis and Mortality in Preterm Infants. *Cell Rep* **14**, 2912-2924 (2016).	Lack of appropriate case and/or control group
Westaway, J. A. F. et al. The Bacterial Gut Microbiome of Probiotic-Treated Very-Preterm Infants: Changes from Admission to Discharge. *Pediatr Res* **92**, 142-150 (2022).	Lack of appropriate case and/or control group
Yang, M. et al. Influence of Family Integrated Care on the Intestinal Microbiome of Preterm Infants with Necrotizing Enterocolitis and Enterostomy: A Preliminary Study. *Front Pediatr* **9**, 678254 (2021).	Lack of appropriate case and/or control group
Young, G. R. et al. Reducing Viability Bias in Analysis of Gut Microbiota in Preterm Infants at Risk of Nec and Sepsis. *Front Cell Infect Microbiol* **7**, 237 (2017).	Did not use next-generation sequencing methods

Table S4. Summary of the certainty of evidence using the GRADE assessment tool.

Number of studies	Risk of bias	Inconsistency	Indirectness	Imprecision	Publication bias	Overall certainty of evidence[a]
Alpha diversity						
24	**Serious/ borderline:** 14 of the 24 studies to assess alpha diversity showed medium or high risk of bias. Most studies showed bias in sampling (subdomains 1 and 2 of the risk of bias) and lacked experimental controls.	**Serious:** findings varied across studies, both in terms of direction of effect and magnitude of effect. Eleven studies reported lower diversity in NEC cases compared to controls, 12 reported no difference and 1 reported higher diversity among NEC cases compared to controls.	**Not serious/ borderline:** the participants (NEC cases and control comparators) provide direct evidence. However, there was substantial variability in the measure/s used to assess alpha diversity. Further the timing of sample collection with respect to NEC diagnosis varied across studies.	**Serious:** the total number of participants across all studies to assess alpha diversity was >1100. However, most studies had a small sample size (<50 participants in n=18/24 studies). The two largest studies to assess alpha diversity reported no association of alpha diversity with NEC.	**Not serious**: publication bias was not suspected because both positive and negative studies were published. Further, only published peer reviewed studies were included, and the systematic search was comprehensive.	low certainty
Beta diversity						
19	**Serious/ borderline:** 11 of the 19 studies to assess beta diversity showed medium or high risk of bias. Most studies showed bias in sampling (subdomains 1 and 2 of the risk of bias) and lacked	**Serious:** findings varied across studies, both in terms of direction of effect and magnitude of effect. Nine studies reported a difference in global microbiota composition between cases and controls, and 10	**Not serious/ borderline:** the participants (NEC cases and control comparators) provide direct evidence. However, there was substantial variability in the measure/s used to assess beta diversity. Further	**Serious:** the total number of participants across all studies to assess beta diversity was >700. However, most studies had a small sample size (<50 participants in n=15/19 studies). The two largest studies to assess beta diversity	**Not serious**: publication bias was not suspected because both positive and negative studies were published. Further, only published peer reviewed studies were included, and the systematic	low certainty

	experimental controls.	studies reported no significant difference.	the timing of sample collection with respect to NEC diagnosis varied across studies.	reported no association of beta diversity with NEC.	search was comprehensive.	
Abundance and/or prevalence of individual taxa						
23	**Serious/ borderline:** 13 of the 23 studies to formally assess individual taxa showed medium or high risk of bias. Most studies showed bias in sampling (subdomains 1 and 2 of the risk of bias) and lacked experimental controls.	**Serious:** findings varied across studies, both in terms of direction of effect and magnitude of effect. No single taxon was associated with NEC in most studies. Eleven studies reported an association between *Enterobacteriaceae* (various genera and species) and NEC, and eight studies reported an association between *Clostridium* and NEC (various species).	**Not serious/ borderline:** the participants (NEC cases and control comparators) provide direct evidence. However, there was substantial variability in the measure/s used to assess the association between NEC and individual taxa. Further the timing of sample collection with respect to NEC diagnosis varied across studies.	**Serious:** the total number of participants across all studies to assess individual taxa was >1100. However, most studies had a small sample size (<50 participants in n=16/23 studies).	**Not serious**: publication bias was not suspected because both positive and negative studies were published. Further, only published peer reviewed studies were included, and the systematic search was comprehensive.	low certainty
